# CD44 targeted functionalized nanocarriers for non-small cell lung cancer

**DOI:** 10.3389/fonc.2025.1692667

**Published:** 2025-11-24

**Authors:** Na Zhu, Ruijuan Guo, Yanyan Jiang, Mingling Xu

**Affiliations:** 1 Medical Oncology Department, Yantaishan Hospital, Yantai, Shandong, China; 2 Department of Pulmonary and Critical Care Medicine, Zibo Central Hospital, Zibo, Shandong, China

**Keywords:** non-small cell lung cancer, CDD44, biomarker, drug delivery, nanocarriers

## Abstract

Non-small cell lung cancer (NSCLC) is a significant worldwide health concern, requiring novel treatment strategies. This review presents the potential of CD44-targeted functionalized nanocarriers as effective tools for treating NSCLC. CD44, a glycoprotein found on surface of cells, is known for being excessively expressed in NSCLC, making it a promising target for targeted drug delivery. The review begins by examining CD44 as a crucial biomarker in NSCLC. The text provides an overview of molecular features of NSCLC. These fundamental concepts provide the framework for comprehending the reasoning behind the focused strategy of medication delivery using nanocarriers. The review discusses the importance of key factors, such as the dimensions, morphology, and electrostatic properties of nanocarriers, in relation to their influence on interactions with CD44 receptors. The review provides an assessment of preclinical and clinical research that has examined the use of CD44-targeted nanocarriers in the treatment of NSCLC. The review further provides an analysis of safety concerns and possible difficulties, like immunogenicity and off-target effects, in relation to CD44-targeted nanocarriers. This review provides helpful guidance to researchers and clinicians who are interested in using CD44-targeted nanocarriers for more precise and efficient therapies of NSCLC.

## 1 Introduction

**Figure 1:**
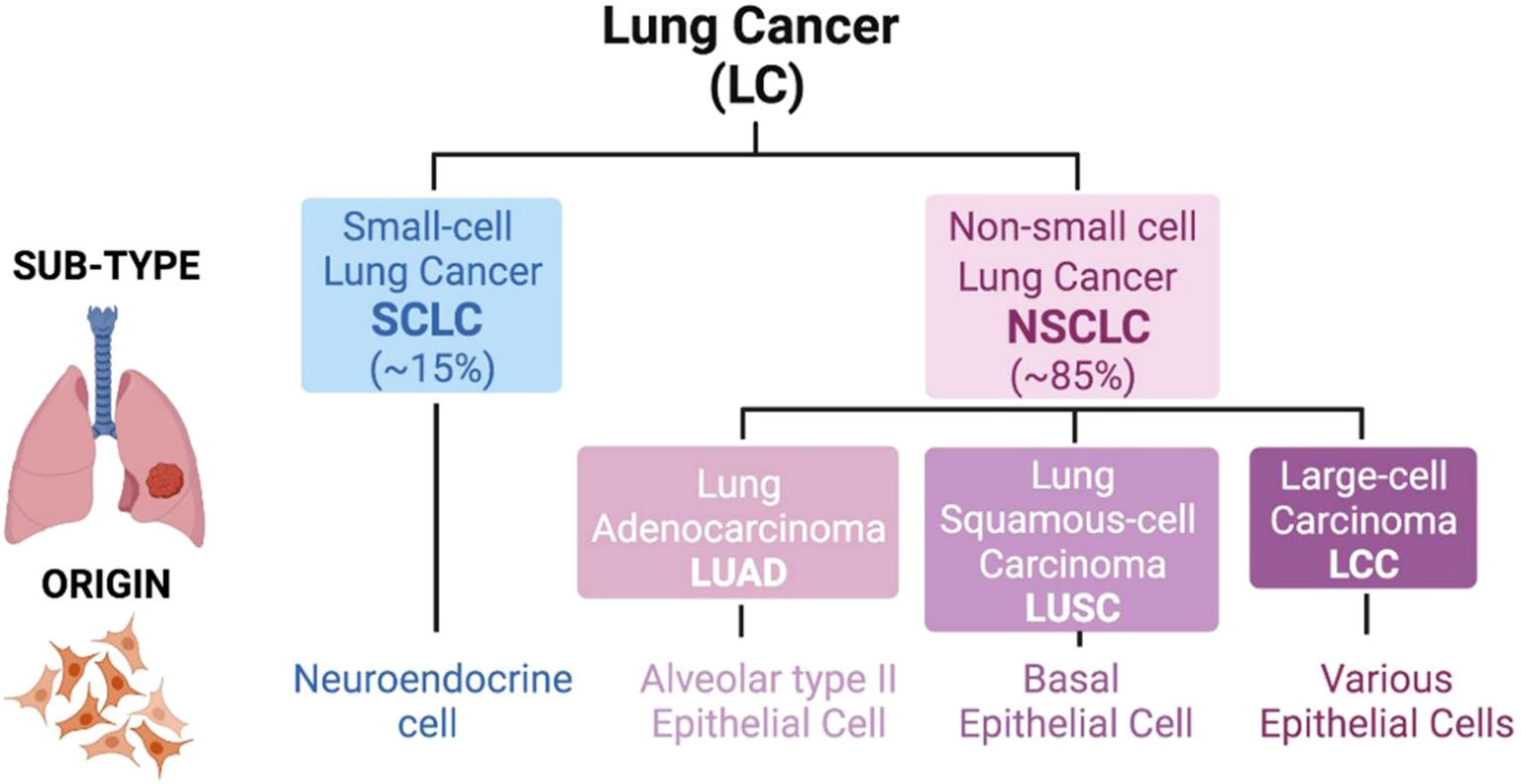
Classification of lung cancer. There are two major types of lung tumors, which are Small-cell Lung Cancer (SCLC; 15% of cases) and Non-small cell Lung Cancer (NSCLC; 85% of cases). An SCLC tumor is based on neuroendocrine cell lining. But in NSCLC the cell of origin of the tumor is varied and the sub-types include Adenocarcinoma (LUAD; alveolar type II epithelial cell), Squamous-cell Carcinoma (LUSC; basal epithelial cell) as well as Large-cell Carcinoma (LCC; various epithelial cells). Adapted from reference (3) under the terms of the Creative Commons Attribution License (CC BY).

Lung cancer is the most common cause of cancer-related deaths globally with 2.5 million new cases and 1.8 million deaths in 2022, representing 12.4% of all cancer cases and 18.7% of all cancer deaths in the world (1). Non-small cell lung cancer (NSCLC) accounts for around 85% of all lung cancer cases, making it the most common type of lung cancer (2), the types of lung cancer are illustrated in **Figure 1**. The disease is most often diagnosed in countries including China, United States and European countries, having the highest incidence rates (4, 5).

Current treatment modalities in treating NSCLC include surgery, chemotherapy, radiotherapy, targeted therapy, and immunotherapy (6). These treatments, however, have some limiting factors. Chemotherapy, for example, is linked to systemic toxicity, and though effective in early disease, it is often only helpful in advanced disease, where drug resistance and propensity to metastasize are common. Targeted therapies such as the use of inhibitors of the epidermal growth factor receptor (EGFR) (7), while they have some advantages, are being hampered by the problem of mechanisms of resistance. Immunotherapies, which have revolutionized treatment of patients with non-small cell lung cancer (NSC), are not universally effective and can cause immune-related adverse effects (8). Furthermore, the prognosis of patients with advanced stage pretty much stage III or IV is poor, with a poor prognosis and life expectancy below 30% of patients (9).

Conventional cancer therapies such as chemotherapy, radiotherapy, surgery and hormone therapy all have their own disadvantages. Chemotherapy, although effective, is often not specific, and acts on both cancerous and healthy cells. This causes systemic toxicity and serious side effects such as nausea, hair loss, fatigue and bone marrow suppression (10). Over time, drug resistance develops which makes it less effective in the long run. Similarly, the radiotherapies although effective in killing the cancerous cells, can also damage the healthy tissues surrounding the cancer, and they may cause side effects such as burning of the skin, tiring of the body and damage to the organs (11). Additionally, it can cause the tumor to come back if it fails to kill all the cancerous cells. Surgery is often used to remove localized tumors but is not always a viable option for tumors in inaccessible spots or in the case of metastatic disease. Moreover, even after surgery, there are chances of recurrence if not all the cancer cells are removed (12). Lastly, hormone therapy is primarily used for hormonal sensitive cancers, such as breast and prostate cancer. But it is also possible for it to lose efficacy as tumors develop resistance over time (13). These conventional methods are limited by their nonspecific targeting, toxicity, and resistance mechanisms, which highlight the need for more targeted therapies that can improve the precision of treatment and minimize side effects. The challenges posed by existing treatment options underscore the pressing need for innovative therapeutic strategies that can improve efficacy, decrease side effects, and overcome resistance. This review is focused on the potential of CD44-targeted functionalized nanocarriers as a promising solution for addressing these unmet needs related to the treatment of NSCLC.

Nanotechnology has become an imminent solution to the challenges of traditional chemotherapy that may be characterized by limited drug specificity, systemic toxicity, and low therapeutic efficacy (14). Drug delivery by use of nanocarriers enables targeted therapy, which improves the specificity of chemotherapeutic compounds against cancer cells with minimal side effects on normal tissues. Some of the typical difficulties with chemotherapy can be overcome through the design of nanocarriers that enhance the bioavailability, stability, and controlled release of chemotherapeutic drugs, including liposomes, polymeric nanoparticle, and dendrimers. Recent nanomedical developments have demonstrated the possibility to not only improve drug delivery mechanism but also mitigate such negative outcomes as nausea, neurotoxicity, and bone marrow suppression that usually accompany chemotherapy (15). Besides, nanotechnology allows integrating therapies, including chemotherapy and immunotherapy, via targeted delivery, which offers a more personalized and efficient method of cancer therapy.

Furthermore, advanced nanocarriers are based on nanotechnology that have been produced to address the problems with traditional chemotherapy (16). Various nanocarriers, including carbon nanotubes, liposomes, polymeric/non-polymeric nanoparticles, nanogels, micelles, and quantum dots, have shown significant promise in delivering chemotherapeutic drugs to specific targets (17).

Nanocarriers have the potential to increase the physicochemical features of chemotherapeutic agents, minimize adverse effects, enable targeted drug delivery, lower medication dosages, prolong blood circulation time, and provide other benefits (18). Nanoparticles (NPs) have the potential to be utilized in passive and active targeting. However, passive targeting has certain limitations. Therefore, it is crucial to develop actively targeting NPs, also known as “intelligent” NPs, which can specifically deliver their cargo to cancerous cells (19). It was previously stated; the molecular composition of cancer cells differs from that of normal cells. Tumor cells exhibit overexpression of several receptors, including transferrin, integrin, folate receptors (FR), sigma, EGFR, and CD44 (17). Hence, the creation of a technology to specifically target these cells might be a means for the effective and dynamic administration of chemotherapeutic drugs.

The biological marker implicated in carcinogenesis should be regarded as a possible prognostic indicator for survival. CD44, a transmembrane glycoprotein that is essential to cell structure, is often used as a marker for cancer stem cells (CSCs). CD44 has been shown to have a significant impact on cancer cell invasion and metastasis, as well as on essential biological processes such as lymphocyte homing, hematopoiesis, inflammation, wound healing, and apoptosis (20).

**Figure 2:**
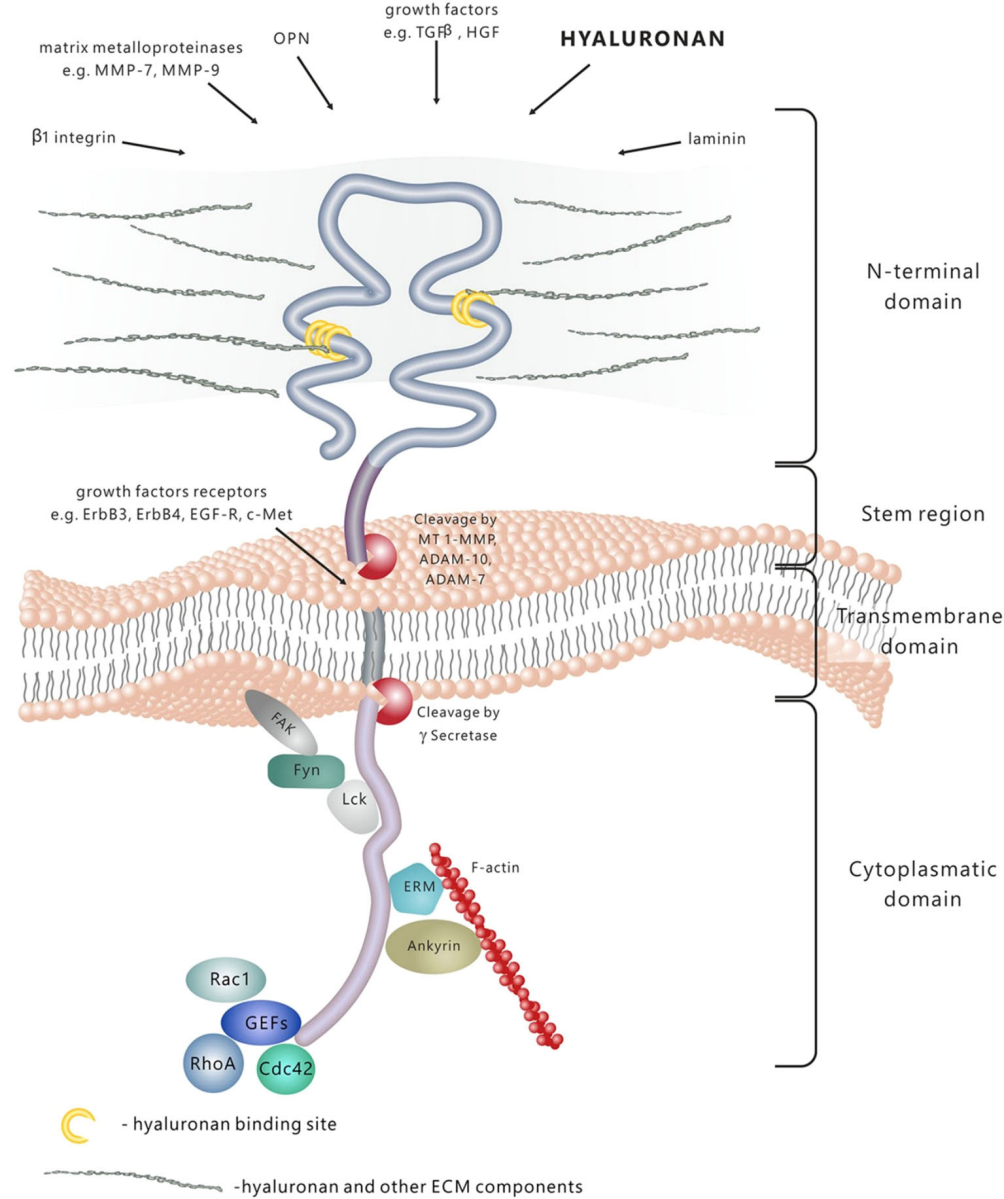
D44 is a transmembrane molecule containing several domains. The N-terminal extracellular domain has the ability of binding a wide range of ligands, among which are hyaluronan (HA), extracellular matrix (ECM) glycoproteins and proteoglycans, growth factors, cytokines and matrix metalloproteins. Due to the proteolytic cleavage in the stem region, the extracellular domain is released in the extracellular space. The transmembrane domain fixes and stabilizes the molecule onto the plasma membrane. Signal transduction is performed by binding to various molecules, such as cytoskeleton elements, small Rho GTPases kinases and activators of guanine nucleotide exchange factors- GEFs. Adapted from reference (21) under the terms of the Creative Commons Attribution License (CC BY). Copyright ^©^ 2015 Dzwonek and Wilczynski.

CD44 has four distinct functional domains, as illustrated in **Figure 2**. The CD44 mRNA undergoes alternative splicing in the proximal extracellular domain, resulting in the generation of several isoforms of CD44. Both the CD44 standard form (CD44s) and CD44 variant (CD44v) are involved in interactions between cells and between cells and the extracellular matrix. They play a role in cell migration, the movement of lymphocytes to specific tissues, and the development of tumors. CD44v6 has garnered more interest in recent years. CD44v6 overexpression has been observed in several types of epithelial malignancies, including head and neck, colon, endometrium, and ovarian cancer. This overexpression likely facilitates the adherence of cancer cells to the vascular endothelium and base membranes, while also increasing the motility of cancer cells (22–24).

CD44 has a crucial role in controlling many important signaling pathways that regulate cancer invasion, metastasis, and resistance to treatment. The modulation of cancer cells is influenced by several substances, including transcription factors, microRNAs, and post-translational changes. CD44 primarily influences cancers by activating signaling pathways that are crucial for apoptosis, epithelial-mesenchymal transition (EMT), and drug resistance (25).

Targeting CD44 has become a potential and feasible approach for treating NSCLC. CD44 has been extensively studied as a flexible therapeutic target in the field of NSCLC (26). The current therapy options include a range of strategies, including neutralizing antibodies, peptide mimetics, aptamers, natural medicines that suppress CD44 synthesis, bioconjugates, and nanoparticles targeted towards hyaluronic acid (HA), HA oligomers, and CD44 decoys (27). Many methods are now being rigorously evaluated in preclinical and clinical settings at various stages, highlighting the ongoing investigation of therapies that target CD44. Several studies have emphasized the significant potential of CD44-targeted nanocarriers in the field of cancer therapy (17, 25, 28). Researchers have identified the CD44 receptor as an important target for anticancers due to its role in regulating tumor cell growth (17). This acknowledgment provides a basis for continuous research, showcasing the progress of scientific studies in converting CD44-targeting approaches into concrete therapeutic progress. Moreover, several research studies have shown fascinating correlations between CD44 and the effectiveness of current treatment options for NSCLC (27). An examination revealed that miR-204, when used, suppresses the proliferation of cancer stem cells and improves the efficacy of osimertinib—a well-established and effective treatment for people struggling with advanced NSCLC (27).

The purpose of this review is to provide a thorough examination of the function of CD44-targeted nanocarriers in the context of NSCLC treatment. The study includes an examination of the biology and clinical importance of CD44 in NSCLC, the progress and promise of CD44-targeted nanocarriers, and relevant information from preclinical and clinical studies. Moreover, the review examines the advantages, challenges, and potential outcomes of using CD44-targeted nanocarriers for NSCLC therapy, aiming to provide information and direction for the advancement of groundbreaking and efficient treatment approaches for this formidable ailment.

## 2 Nano carriers in cancer therapy

Nanocarriers have become revolutionary instruments for transporting anticancer medications, providing a varied and advanced method to tackle crucial obstacles in cancer treatment (29). Nanocarriers include a wide variety of materials and structures, including chemical and inorganic molecules, as well as lipid and protein-based entities. These entities generally have sizes ranging from 1 to 100 nm (30–32).

Nevertheless, nanocarriers demonstrate their success through many of their properties including size, surface charge, drug loading, and release, which are not consistently native and can only be effectively changed through design alterations. Passive targeting of nanocarriers is most effective at 10–100 nm, which is the size of the nanocarriers, which are identified as nanocarriers, but to maximize the ability to target tumors the size of nanocarriers needs to be regulated. Among the design strategies, it is possible to use materials that can be fabricated in the desired size (such as PLGA or lipid-based carriers), and alter the shape of nanoparticles (e.g., rod-like) to enhance cellular uptake and accumulation in tumors (33). Also, the surface charge can be engineered to enhance cell membrane interaction or reduce immune detection and surface functionalization with ligands (e.g. antibodies and peptides) can be used to target the particles to cancer cells. These systems are biocompatible and stable by using techniques such as PEGylation to increase circulation time and decrease immunogenicity (34).

Nanocarriers determine their drug loading and release through the material composition and addition of tailored alterations to achieve controlled drug release. Drugs can be delivered at the tumor site specifically with the help of stimuli-responsive nanocarriers, including pH-sensitive polymers, which reduce systemic toxicity. High drug payload and controlled release is often used through liposomes and polymeric micelles (35). Biocompatibility and stability play an important role as well, with nanocarriers frequently being made of biodegradable materials to reduce the number of toxic proteins, as well as degrade safely in the body. Passive and active mechanisms increase tumor targeting with nanocarriers that are functionalized with special ligands binding to overexpressed receptors on the tumor cells. This directional targeting enhances specificity of drug delivery with minimal effects being done on normal tissues. Moreover, it is important to overcome the multidrug resistance (MDR), and nanocarriers may be designed to circumvent the efflux pumps by co-delivering drugs with MDR inhibitors, enhancing the activity of chemotherapy against drug-resistant cancer (36, 37). These design principles empower nanocarriers to respond to the specific problem of cancer treatment and achieve excellent treatment results. Moreover, each nanocarrier has distinct characteristics that are carefully adjusted for drug delivery purposes. This enables the encapsulation and precise release of therapeutic substances to cancer cells, hence improving the accuracy of treatment (38).

Nanocarriers increase the bioavailability and solubility of pharmaceuticals, resulting in enhanced pharmacokinetics and decreased adverse effects. The enhanced solubility guarantees a more efficient delivery of therapeutic drugs to cancer cells, hence augmenting the overall efficacy of the treatment (39, 40). They provide the precise targeting of cancerous tissues or cells, allowing for selective treatment and minimal harm to healthy cells. This tailored technique guarantees that therapeutic drugs are supplied exactly to the location of the tumor, maximizing the therapeutic ratio (41, 42). Nanocarriers enable the regulated and extended release of medications, ensuring that cancer cells are consistently exposed to therapeutic payloads. The sustained release mechanism prolongs the therapeutic impact, which may lead to a decrease in the frequency of administration and an improvement in patient compliance (16, 43). They have a role in diminishing the harmful effects on cells and improving the absorption of medications by cancer cells (44). This not only enhances the overall therapeutic effectiveness but also reduces unintended effects on non-target areas, therefore tackling a major obstacle in traditional cancer therapies (41). Moreover, nanocarriers have shown the ability to surmount multidrug resistance in cancer cells, representing a noteworthy advancement in tackling a prominent obstacle in chemotherapy (45). This characteristic presents novel opportunities for augmenting the efficacy of current anticancer medications (46).

## 3 Cancer therapy using CD44-engineered nanocarriers

**Figure 3:**
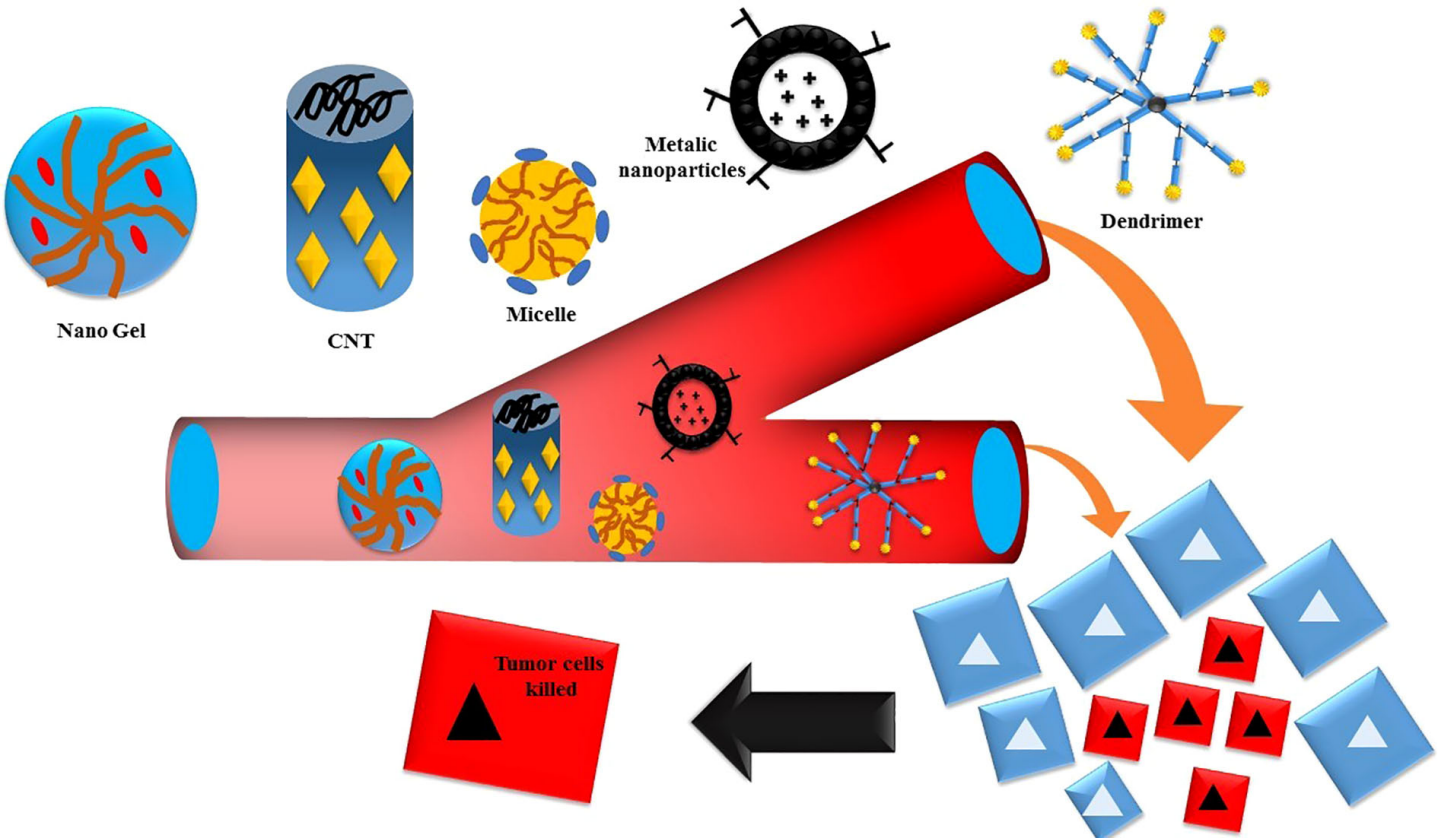
Diagram depicting the targeted delivery of anticancer medicines using CD44.

Nanoparticles (NPs) that have a strong attraction to the CD44 receptor can deliver therapeutic substances to certain cells in a targeted manner while limiting any negative effects on normal cells (47). Enhancing absorption and inducing apoptosis may be achieved by modifying the nano-drug delivery system by surface attachment of chemicals. Nanocarriers have many benefits, such as easy production, large drug load capacity, greater solubility, improved drug availability, efficient drug dispersion in the body, higher permeability across physiological barriers, and decreased drug toxicity (48), illustrated in **Figure 3**.

### 3.1 HA-modified nanocarriers for drug delivery

Muntimadugu et al. conducted a study where they improved the delivery of anticancer medications targeted at CD44 by utilizing a nanoparticle made of HA-coated PLGA. The study demonstrated higher effectiveness in trapping the drugs and increased cytotoxicity in MCF-7 cells, which was dependent on the concentration of the pharmaceuticals (49). Additional research conducted by Saneja et al. and Liu et al. provides evidence of the efficacy of ligand-modified PEGylated PLGA nanoparticles in attaining precise drug distribution and increased cellular uptake (50–52). HA-modified nanoparticles have been used for the delivery of several anticancer drugs, such as thio-tetrazoly analogues, 10-hydroxy camptothecin, and green tea epigallocatechin-3-gallate (53, 54). In addition, Shi et al. and Lee et al. have developed dual-targeting systems that combine HA with other ligands. These systems have shown encouraging outcomes in the active targeted delivery of chemotherapeutic drugs (53–55).

### 3.2 Metallic and ceramic nanocarriers

Metallic nanoparticles, including magnetic iron oxide (Fe3O4) and AuNPs, have attracted considerable interest due to their distinctive characteristics in the field of biological applications. The study done by Sargazi et al. demonstrates the efficacy of HA-conjugated PEGylated MNPs for targeted delivery of mitoxantrone to CD44 (56–58). Moreover, the use of Fe3O4@Ag-HA-NSs in multifunctional theranostic methods has shown promise in the fields of cancer diagnostics and photothermal treatment (56).

For example, there is a case study which focuses on the creative use of folic acid-modified PEGylated paramagnetic nanoparticles as drug delivery vehicles that specifically target CD44. Folic acid, known for its strong attraction to cancer cells, particularly those that have an excessive amount of CD44 receptors, is deliberately selected as the ligand for the surface of the nanocarrier. This ligand promotes receptor-mediated endocytosis, which guarantees selective absorption by cancer cells that express CD44. The use of polyethylene glycol (PEG) has a double effect—it improves the stability and biocompatibility of the nanoparticles while extending their duration in the bloodstream. PEGylation enhances the nanoparticles’ half-life and reduces non-specific interactions, leading to improved biodistribution and targeted accumulation at the tumor location (59).

The experimental results obtained from investigations conducted on NSCLC cell lines provide solid evidence for the efficacy of this strategy. The cellular absorption of folic acid-modified PEGylated paramagnetic nanoparticles shows a significant increase when compared to their non-targeted counterparts. The nanoparticles possess paramagnetic properties, which allow for their real-time tracking by magnetic resonance imaging (MRI), adding an extra level of usefulness. This imaging capacity offers vital information into the geographical and temporal dynamics of the nanocarrier inside the tumor microenvironment (59).

This nanocarrier that targets CD44 has a significant influence on the therapeutic process. The loaded therapeutic payload, which often consists of powerful anticancer medications like paclitaxel or doxorubicin, demonstrates increased effectiveness because of the accuracy of targeted administration. This multifarious CD44-targeted nanocarrier has the potential to be useful in therapeutic settings, as shown by the case study, which indicates that it inhibits the development of tumors in preclinical models of non-small cell lung cancer. This method is a potential route in the search for effective therapies for NSCLC because it integrates imaging capabilities, targeted drug delivery, and increased therapeutic results from a therapeutic perspective (59).

Ceramic nanoparticles, particularly mesoporous silica nanoparticles (MSNs), have been investigated for their distinct characteristics in delivering drugs, demonstrating combined anticancer benefits against colon cancer (60). Nanoparticle-based drug delivery systems, namely those enhanced with HA for precise targeting, provide a hopeful approach in the field of cancer treatment. Due to their passive tumor targeting and tiny size, nanoparticles provide distinct benefits for effective and regulated drug administration (61).

### 3.3 Carbon nanotubes and other nanocarriers

In 1991, Iijima made the pioneering observation of carbon nanotubes (CNTs), tubular carbon structures, with two primary types: single-walled carbon nanotubes (SWCNTs) and multi-walled carbon nanotubes (MWCNTs) (62). CNTs exhibits remarkable properties such as excellent electrical conductivity, ordered structure, low weight, and thermal conductivity, making them potential candidates for various applications, including biomedical. The unique needle-shaped structure of CNTs enables them to easily penetrate the plasma membrane and directly reach targeted cells (63). This, coupled with their targeted and controlled drug delivery capabilities, positions them as promising carriers for therapeutic purposes. The surface versatility of CNTs permits conjugation with different targeting ligands, making them particularly suitable for CD44 receptor-targeted drug delivery in cancer treatment (64). Their one-of-a-kind tubular shape, carbon nanotubes provide an unrivaled surface area, making them a perfect platform for the loading and functionalization of medical drugs. When it comes to CD44 receptors, HA, which is a naturally occurring ligand, is selected because of its specificity and affinity for these receptors (65). This design is based on several different rationales: first, the tubular structure of carbon nanotubes makes it possible to load therapeutic agents efficiently; second, the functionalization of the nanocarrier with HA ensures that it will selectively bind to CD44 receptors, which makes it easier to deliver specifically targeted drugs to cancer cells. By *in vitro* tests, the effectiveness of this nanocarrier design is thoroughly measured and evaluated. The results of these experiments demonstrate that CD44-positive non-small cell lung cancer cells can effectively internalize HA-functionalized carbon nanotubes. The unique interaction that occurs between HA and CD44 receptors leads to an increase in the cellular absorption of the nanocarrier, which in turn leads to an increase in the concentration of the medication inside the cell. When it comes to obtaining accuracy in medication delivery and reducing off-target effects, this targeted internalization is an essential step that must ultimately be taken (65).

Furthermore, the outcomes of the experiments shed insight into the drug release kinetics of the carbon nanotubes that have been functionalized with amino acids (HA). Not only can the functionalization with HA improve targeting, but it also affects the regulated release of medicinal drugs. The presence of this regulated release profile indicates that there is the possibility of long-term therapeutic benefits. One of the benefits of the progressive and continuous release of medications is that it may contribute to the extended exposure of cancer cells to the therapeutic payload, which ultimately results in the enhanced effectiveness of the treatment. To gain useful insights into the potential clinical uses of HA-functionalized carbon nanotubes, the therapeutic effect of these nanotubes is evaluated using models of NSCLC (65). In comparison to their non-targeted equivalents, the evidence of lower systemic toxicity is a discovery that deserves noteworthy attention. By demonstrating a higher degree of selectivity, the nanocarriers that have been functionalized with HA make it possible to deliver drugs to cancer cells with more precision. This tailored method reduces the possibility of systemic toxicity by minimizing the exposure of healthy tissues to the therapeutic payload to limit the therapeutic payload. When it comes to improving the safety profile of the nanocarrier and its potential for clinical translation, achievements of this kind are very essential (65).

Carbon nanotubes are a versatile substrate for CD44-targeted drug delivery, as shown by this case study, which highlights their adaptability. Carbon nanotubes are attractive prospects in the field of nanomedicine due to their distinctive structural properties, which, when combined with the selective binding that is made possible by HA functionalization, position them as very promising possibilities. The capability of the nanocarrier to load medications effectively, improve cellular absorption, and give regulated drug release are all examples of the many benefits that this technique offers (65). For CD44-targeted drug delivery, the functionalization of carbon nanotubes with hyaluronic acid offers a complex technique that has intriguing implications for the treatment of NSCLC (65). The outcomes of the experiments provide evidence that this method is feasible. They highlight the potential therapeutic benefit of this strategy, as well as its decreased systemic toxicity and the adaptability of carbon nanotubes as an excellent platform for targeted drug delivery in cancer therapies. Targeting CD44 has emerged as a feasible approach for NSCLC treatment, with various strategies like neutralizing antibodies, peptides, aptamers, natural medicines, bioconjugates, and nanoparticles. CD44-targeted nanocarriers have garnered attention for their potential in cancer therapy (66). Studies utilizing HA-functionalized CNTs exemplify this approach.

Singhai et al. employed MWCNTs functionalized with HA and α-Tocopheryl succinate (α-TOS) for targeted doxorubicin (DOX) delivery against triple-negative breast cancer (TNBC) cells. The formulation exhibited high drug loading, and *in vitro* cytotoxicity results demonstrated superior efficacy, showcasing the potential of CD44-targeted nanocarriers (67). Additionally, nanoemulsions (NEs) and micelles, as nanocarriers, were explored for targeted drug delivery by incorporating HA, showing enhanced tumor reduction and improved pharmacokinetic profiles (68–71). Quantum dots (QDs) modified with HA emerged as effective tools for bioimaging tumor cells, showcasing their potential in diagnostic applications. The enhanced biocompatibility and CD44 receptor-mediated cellular internalization demonstrated the promising role of HA-modified QDs in targeted drug delivery (72, 73).

Nanogels (NGs) are advanced nanocarriers made from hydrogel and cross-linked hydrophilic polymers, with the potential to drug delivery to cancer cells. They offer enhanced functionality, ease of formation, improved release capability, superior targetability, and customizable attributes. Wei and colleagues developed NG drug conjugates based on hyaluronic acid (HA) for targeted delivery of etoposide (ETO), salinomycin (SAL), and curcumin (CUR). These formulations showed passive accumulation in tumor cells through enhanced permeability retention. *In vitro* cytotoxicity studies showed promising IC50 values for CHA-ETO, CHA-SAL, and CHA-CUR, surpassing those of free drugs. Cholesterol moieties further improved tumor accumulation, leading to enhanced drug bioavailability and therapeutic efficacy against resistant cancer cells (74–76).

SiRNAs, due to their potent gene-silencing effects, hold promise for treating various diseases, including cancers. However, their clinical application faces challenges such as poor cellular penetration, instability in biological fluids, and lack of targetability. To address these issues, nanocarriers have been designed, focusing on non-viral vectors like cationic liposomes, polymers, and dendrimers. CD44-engineered nanocarriers, particularly those incorporating HA as a targeting ligand, have shown efficacy in delivering siRNAs (77). Yoon et al. developed a biodegradable HA cross-linked poly (dimethyl amino ethyl methacrylate) HPD conjugate for siRNA delivery. The cross-linked siRNA-HPD complex demonstrated stable formation via disulfide bonds, with *in vitro* cytotoxicity studies showing a significant decrease in B16F10 cell viability.

**Figure 4:**
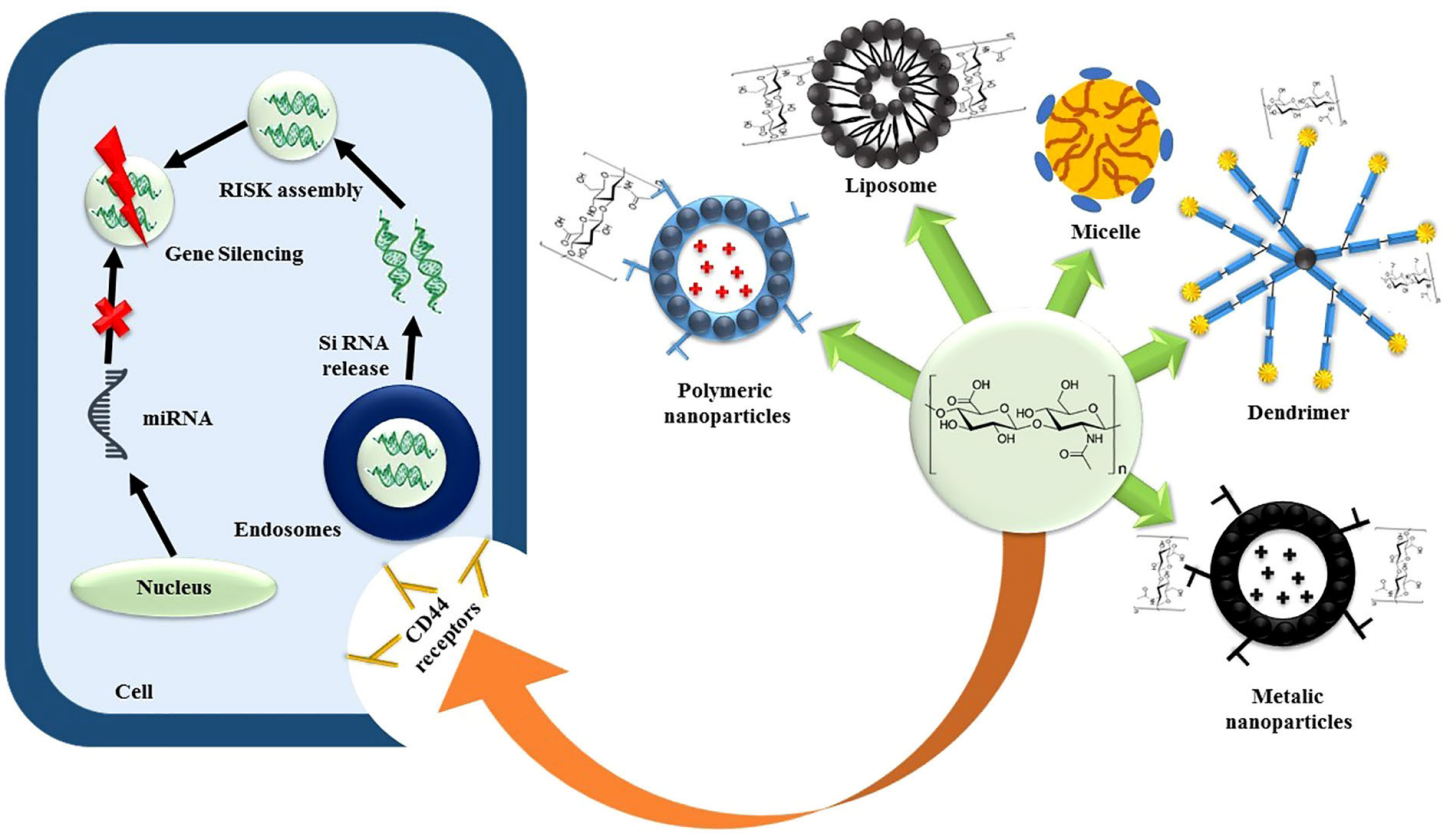
The schematic representation of C44-mediated SiRNA delivery.

Cellular uptake and gene silencing studies supported the efficient CD44-mediated endocytosis of the cross-linked siRNA-HPD complex (78). Shah et al. designed a CD44-targeted nanocarrier for co-delivery of siRNA and the anticancer drug paclitaxel (PTX) in ovarian cancer. The formulation involved linking PTX to PPI dendrimers via succinic acid, conjugating luteinizing hormone-releasing hormone (LHRH) peptide as the targeting ligand to PEG polymers, and complexing PPI dendrimers with siRNA (PPI-siRNA) (79), the schematic representation is illustrated via **Figure 4**. Herrera et al. utilized ternary and quaternary polyplexes consisting of siRNA-bPEI modified with glycosaminoglycan (GAG) polysaccharides (HA, CS, and HA) for silencing green fluorescent protein expression in human mesenchymal stem cells (hMSCs). Xiong et al. designed a codelivery system for the anticancer drug DOX and a gene decorated with HA against hepatocellular carcinoma (80).

Chondroitin sulfate (CS)-modified nanocarriers have shown significant promise in cancer therapy. Recent studies have reported the development of docetaxel-loaded Zein nanoparticles modified with CS for effective treatment against prostate cancer, temozolomide-loaded albumin nanoparticles for brain-targeted delivery, and CS and HA nanoparticles loaded with doxorubicin for effective therapeutic outcomes (81–83).

## 4 Enhancing personalized treatment NSCLC with nanocarriers that target CD44

The future of cancer treatment is shifting towards personalized medicine and CD44-targeted nanocarriers have become an excellent option in the customization of interventions in non-small cell lung cancer (NSCLC) patients. Recent research outlines the opportunities of biomarker profiling e.g., genetic mutations and protein expression levels to inform the individualized use of CD44-targeted treatment. The CD-44 targeted nanocarriers used for personalized treatment of NSCLC are given in **Table 1**. To illustrate, Wickens et al. (2017) revealed that NSCLC patients who were highly expressing CD44 responded more to HA-conjugated nanoparticles, which underscored the need to profile patients with biomarkers to respond to these therapies (28). The presence of biomarkers such as CD44 receptor isoforms, genetic mutations, or protein overexpression is very helpful to determine the heterogeneity of tumors and to design more effective and less side effects treatment approaches (17, 25, 90).

**Table 1:** Strategies for personalized treatment of NSCLC using CD44-targeted nanocarriers.

Nanocarrier type	Targeting ligand	Drug/RNA payload	Size (nm)	Administration route	Mechanism of CD44 targeting	Outcomes/advantages	Patient-specific variables	Reference
Hyaluronan-based nanoparticles	Hyaluronic acid	Imaging agents	80, 200	IV, inhalation	HA-CD44 mediated endocytosis; size impacts tumor uptake	Improved tumor accumulation and active CD44 targeting	Tumor CD44 expression, pulmonary function, immune status	(84)
Biodegradable PCL-CP nanocarrier	Chondroitin sulfate, PEG	Doxorubicin + pre-siiRhom (siRNA for iRhom1)	~110	IV	CS-CD44 interaction enables endothelial/tumor cell transcytosis and targeting	Enhanced cytotoxicity, immune activation, and lower resistance	iRhom1 status, immune checkpoint profile, CD44 isoform, chemo history	(85)
Indocyanine green-PTX-HSA nanoparticles	Hyaluronic acid	Indocyanine green, Paclitaxel	Not reported	IV	HA targets CD44-positive NSCLC via ligand-receptor specificity	Targeted imaging and dual therapy capability	Tumor molecular subtype; CD44 positivity, imaging requirements	(86)
HA-targeted topotecan liposomes	Hyaluronic acid	Topotecan	~98	IV	Surface HA enables CD44 binding and endocytosis	Greater uptake, prolonged circulation, stronger tumor inhibition	Topotecan sensitivity, baseline liver function, tumor CD44 status	(87)
HA-modified HSA-Paclitaxel/ICG nanoparticles	Hyaluronic acid	Paclitaxel, Indocyanine green	~100	IV	HA coating boosts CD44-mediated tumor accumulation	Effective for image-guided chemotherapy	Tumor CD44, biodistribution needs, drug resistance profile	(88)
HA-NP cisplatin/siRNA delivery	Hyaluronic acid	siRNA duplexes, cisplatin	Not stated	IV	HA improves tumor accumulation and efficient delivery to tumor via CD44	Enhanced efficacy in combination treatments	Genetic variants, cisplatin resistance, CD44 status	(89)
PLGA SLM/PTX nanoparticles	Hyaluronic acid	Salinomycin, Paclitaxel	Not reported	IV	HA-mediated CD44 targeting, improved cancer stem cell elimination	Lower IC50, more selective CSC targeting	Tumor stemness profile, CD44 level, prior stem cell-directed therapy	(84)

Including genetic and molecular profiling would be essential in screening patients that would respond to CD44-targeted nanocarrier therapies. Researchers like Tirella et al. (2019) have found that patients with mutations in EGFR or KRAS respond differently to therapy using CD44-targeted nanocarriers (52). The design of nanocarriers fit the specifics of each tumor is another important factor. As an example, nanocarriers can be tailored to more accurately target individual CD44 isoforms or adapt to differences in tumor microenvironment, which would guarantee improved delivery of drugs and effective treatment (17). In their study, Limpikirati et al. (2025) demonstrated that adjusting the size and surface properties of nanocarriers, depending on the vascular architecture of the tumor and the level of CD44 expression, enhanced the targeting ability of HA-conjugated nanoparticles to NSCLC models (91).

Additionally, individual patient factors, including renal status, tumor mass, and immune condition are important determinants of nanocarrier efficacy of CD44-targeted nanocarriers. The therapeutic effect of nanocarriers can be increased by adjusting the pharmacokinetics and clearance rates of nanocarriers according to these individual characteristics. Adapting the design of nanocarriers according to specific patient features is vital progress in customizing therapy for NSCLC (92). The effectiveness and safety of CD44-targeted nanocarriers may be greatly influenced by individual patient characteristics, such as the tumor microenvironment, genetic composition, and general health condition. Multiple recent research has examined the notion of customized nanocarrier design (93–95). For example, knowing the particular CD44 isoforms or expression levels in a patient’s tumor might help in adjusting nanocarriers to improve their ability to attach and enter cells (96).

Furthermore, taking into account the distinct physiological characteristics of each patient, such as renal function or clearance rates, enables the enhancement of nanocarrier qualities, guaranteeing a customized and optimum treatment strategy (97). The individualized strategy for designing nanocarriers has shown encouraging findings in preclinical models, showcasing enhanced therapeutic efficacy and decreased off-target effects. Through the alignment of nanocarrier attributes with the specific biological and physiological traits of each patient, medical practitioners may optimize treatment accuracy, possibly resulting in improved therapeutic outcomes and fewer negative impacts. The incorporation of biomarker analysis and the tailoring of nanocarrier design according to patient characteristics are crucial advancements in the progression of tailored treatment for NSCLC (98). CD44-targeted nanocarriers may be customized to optimize effectiveness and minimize adverse effects by leveraging the distinct characteristics of each patient’s cancer (85). This advancement paves the way for a revolutionary age of precision medicine in the treatment of NSCLC. Further investigation and rigorous clinical validation are necessary to fully exploit the promise of this individualized therapy strategy.

## 5 Immunotherapeutic uses of CD44-targeted nanocarriers in NSCLC

CD44-targeted nanocarriers are emerging as flexible challengers in the field of cancer therapies, namely in the promising area of immunotherapy for lung cancer (99). In addition to its traditional function, CD44 also displays inherent immunogenicity, which offers a promising opportunity to enhance the body’s immune response against NSCLC (100). To strategically exploit the immunogenic features of CD44, it is necessary to create nanocarriers that can transport therapeutic payloads and also actively regulate immune responses inside the tumor environment (100, 101). CD44-targeted nanocarriers seek to enhance the antitumor immune response by either including immunostimulatory chemicals or facilitating the activation of immune cells (102). This novel strategy shows potential for promoting a more extensive and long-lasting therapeutic impact, guiding the treatment of NSCLC towards a strong immunotherapeutic model. Recent literature has been conducted which shows that CD44 targeted nanocarriers can enhance the delivery and effectiveness of checkpoint inhibitors, including anti-PD-1, by specifically targeting CD44-overexpressing tumor cells which are frequently resistant to traditional immune checkpoint blockage treatments (17, 103–105). These nanocarriers increase the penetration of immune cells into the tumor microenvironment, especially cytotoxic T lymphocytes (CTLs), therefore increasing the anti-tumor immune response (106).

Simultaneously, the nanocarriers are cleverly designed to act as immunomodulatory agents in NSCLC (107). Incorporating immune-modulating drugs directly into the nanocarrier design is the novel technique that is being taken here. Recent studies look at the possibility of incorporating immunomodulatory chemicals, such as checkpoint inhibitors or cytokines, into nanocarriers that are particularly designed to target CD44 (108). In addition to checkpoint inhibitors, other immune stimulating agents such as interleukins (e.g., IL-2, IL-12) or tumor necrosis factor-alpha (TNF alpha) have been incorporated into the CD44-targeted nanocarriers to boost T-cell activation and attraction of immune effector cells to the tumor site (109–112). This combination of immune modulators and nanocarriers has been shown to synergically enhance the immune response in models of NHL suggesting increased endogenous tumor suppression and more durable response. To combat the immunosuppressive circumstances that are characteristic of NSCLC, the objective is to actively manage immune responses. When immunomodulatory medicines are combined with nanocarriers that are targeted to CD44, the goal is to improve the effectiveness of immunotherapy in NSCLC, hence supporting a therapeutic plan that is unified (107).

Engineering advancements in CD44-targeted nanocarriers expand into the realm of biomimicry, creating platforms that closely imitate the inherent traits of NSCLC cells. Biomimetic nanocarriers, which draw inspiration from the complex biological architecture, improve their interaction with cancer cells that overexpress CD44 (113). Moreover, the emergence of new designing of nanocarriers is in the direction of biomimetic platforms which mimic the natural characteristics of tumor cells. These biomimetic nanocarriers, which mimic the lipid make-up and surface properties of the cell membrane of lung cancer, have been shown to have enhanced cellular affinity for CD44 cancer-overexpressing cells, leading to better uptake and immune activation (114). The biomimetic nature of these nanocarriers makes them better at avoiding being detected by the immune system and guaranteeing good delivery to the site of the tumor. Recent research has shown that biomimetic nanocarriers are very effective in enhancing the accuracy of targeting and the absorption of cells (115). These biomimetic devices improve the identification and absorption of therapeutic payloads by imitating the characteristics of NSCLC cell membranes, which might improve treatment results. This sophisticated technique indicates a shift away from traditional ways of administering drugs, adopting a more customized and individualized manner (115). Responsive nanocarriers revolutionize medication delivery by providing adaptable modifications that respond to the unique requirements of the NSCLC microenvironment (116). The advanced nanocarriers adjust their drug release patterns in response to stimuli like as pH, enzyme activity, or hypoxia, which are often seen in NSCLC tumors (116, 117).

## 6 Mechanisms of action and comparison

### 6.1 Mechanisms of action of CD44 targeted functionalized nanocarriers

The fundamental basis of the method is the precise contact between CD44 receptors, which are highly expressed on the surface of cancer cells, and the ligands that are integrated into the nanocarriers (118). Ligands, such as HA, antibodies, peptides, or folic acid, have a crucial function. This connection promotes specific attachment and uptake of the nanocarriers into cancer cells that express CD44, allowing for precise delivery of drugs (119). Upon binding to CD44 receptors, the nanocarriers undergo receptor-mediated endocytosis, which guarantees their internalization into the cancer cells (120). Inside the cellular environment, the therapeutic cargo, which might consist of anticancer medications such as paclitaxel or doxorubicin, is released. The internalization selectivity guarantees the targeted delivery of the therapeutic payload only to cancer cells, hence reducing its exposure to healthy tissues (121). Paclitaxel (PTX), a frequently used antineoplastic drug, is included in the nanoparticles. The primary objective in providing PTX to NSCLC is to get a knowledge of the therapeutic impact of CD44-targeted nanoparticles (86). The design revolves around the incorporation of ligands, such as antibodies or peptides, onto the surface of the nanoparticles that exhibit great specificity towards CD44. Due to this deliberate choice, the nanocarrier may specifically bind to CD44 receptors that are present on the outer layer of cancer cells. To showcase the potential of CD44-targeted nanoparticles in delivering traditional chemotherapeutic medicines, PTX is used as a representative therapeutic payload (86).

This case study is based on extensive *in vitro* research that examines the behavior of nanoparticles specifically targeted at CD44 in a controlled biological setting. Based on the results of these inquiries, it has been shown that non-small cell lung cancer cells have a predilection for internalizing nanoparticles that are specifically designed to target CD44. This offers compelling evidence of the nanocarrier’s specificity. An extensive examination of cellular internalization mechanisms is underway to gain insight into the molecular connections between nanoparticles and cancer cells. Furthermore, the study assesses the kinetics of drug release, providing insights into the controlled release pattern of PTX from the nanoparticles. Considering all of this evidence, it is evident that nanoparticles specifically designed to target CD44 can greatly enhance the transportation of therapeutic medications to cancer cells (86). Transitioning from *in vitro* assessments to *in vivo* studies is a crucial step in assessing the therapeutic value of CD44-targeted nanoparticles (86). Throughout this case study, *in vivo* studies were conducted, and the findings demonstrated a significant therapeutic effect. The efficacy of CD44-targeted nanoparticles encapsulating PTX is shown by the efficient inhibition of tumor growth. Additional proof of the potential therapeutic efficacy of these nanoparticles is shown by their ability to enhance survival rates in NSCLC models upon treatment. The efficacy and versatility of CD44-targeted nanoparticles in cancer treatments are shown by their capacity to effectively deliver conventional chemotherapeutic agents such as PTX (86).

Targeted administration of therapeutic drugs to cancer cells using CD44-specific nanocarriers has a role in suppressing tumor development and metastasis. Through the manipulation of the signaling pathways linked to CD44 expression, these nanocarriers have the potential to hinder the aggressive activity of cancer cells, therefore inhibiting their capacity to multiply and move (96). CD44 is involved in drug resistance mechanisms, namely in cancer stem cells (122). Nanocarriers that specifically target CD44 have shown the potential to surmount this resistance by directly delivering therapeutic medicines to these populations of drug-resistant cells (123). CD44 is recognized for its involvement in several signaling pathways, including those related to cell viability, growth, and movement (124). CD44-targeted nanocarriers can manipulate these pathways, interrupting the complex cellular processes that play a role in the advancement of cancer. This modification may result in a decrease in the malignancy of cancer cells and make them more responsive to treatment therapies (125). CD44 has a role in modulating the immune system by influencing processes including the recruitment and activation of immune cells (126). CD44-targeted nanocarriers might potentially impact the immune response to cancer by regulating the interactions between cancer cells and immune cells. The combination of directly attacking cancer cells and regulating the immune response may synergistically boost the total anticancer impact (17).

### 6.2 Comparison with other targeted therapies in NSCLC treatment

The addition of CD44-targeted functionalized nanocarriers into the field of NSCLC therapy necessitates a detailed evaluation in contrast to existing targeted treatments, highlighting unique characteristics and possible collaborative effects (127), the comparison is also mentioned via the **Table 2**. Unlike treatments that focus on single molecular markers, CD44-targeted nanocarriers use a complete targeting strategy that encompasses all aspects of cancer development, going beyond individual indicators (128). Due to their adaptability, these treatments have the potential to effectively address several elements of NSCLC, setting them apart from therapies that focus on particular molecular targets (129).

**Table 2:** Comparison of CD44 treatment with other targeted therapies in NSCLC.

Therapy type	Target	Mechanism	Advantages	Limitations	Current status in NSCLC
CD44-Targeted Nanocarriers	CD44 receptor on tumor cells	Nanocarriers targeting CD44 with conjugated therapeutic agents (chemotherapeutic agents, checkpoint inhibitors)	1. Tumor-specific targeting 2. Reduced systemic toxicity 3. Potential for combination with immunotherapy	1. Tumor heterogeneity in CD44 expression 2. Limited clinical data on long-term efficacy 3. Potential resistance mechanisms	Early-stage preclinical studies, ongoing clinical trials investigating combination with chemotherapy and immunotherapy
EGFR Inhibitors (e.g., Gefitinib, Erlotinib)	EGFR mutation in tumor cells	Small molecules that block EGFR signaling, inhibiting tumor growth	1. Effective in EGFR-mutant NSCLC 2. Improved survival in specific EGFR-positive patients	1. Acquired resistance (e.g., T790M mutation) 2. Limited to EGFR-mutant tumors 3. Off-target toxicity	Widely used in EGFR-mutant NSCLC, but resistance is common
ALK Inhibitors (e.g., Crizotinib)	ALK gene rearrangement in tumor cells	Small molecules that target and inhibit the activity of ALK gene rearrangement in NSCLC	1. Effective for ALK-positive NSCLC 2. Better response rates in ALK-positive patients	1. Acquired resistance (e.g., secondary mutations) 2. Limited use to ALK-positive tumors 3. Systemic side effects	Approved for ALK-positive NSCLC, but resistance can develop with prolonged use
Immunotherapy (e.g., PD-1/PD-L1 Inhibitors, Nivolumab)	PD-1/PD-L1 interaction in immune cells	Blockade of immune checkpoint proteins to enhance T-cell response against cancer cells	1. Long-term survival benefits in PD-L1-positive tumors 2. Potential for combination with other therapies	1. Low response rates in cold tumors 2. Limited benefit for non-squamous subtypes3. Immune-related adverse events	FDA-approved for NSCLC treatment, but low efficacy in certain subsets of patients
VEGF Inhibitors (e.g., Bevacizumab)	Vascular endothelial growth factor (VEGF)	Inhibit VEGF to reduce tumor angiogenesis and improve oxygen delivery	1. Effective in combination with chemotherapy 2. Improves tumor perfusion and drug delivery	1. High cost2. Potential for serious side effects like bleeding or hypertension	Used in combination with chemotherapy, especially in non-squamous NSCLC
KRAS Inhibitors (e.g., Sotorasib)	KRAS mutations in tumor cells	Small molecules that specifically inhibit mutant KRAS, commonly found in NSCLC tumors	1. Effective in KRAS-mutant NSCLC 2. Targets previously “undruggable” mutations	1. Limited to KRAS-mutant tumors 2. Resistance may develop over time	FDA-approved for KRAS G12C-mutant NSCLC, but challenges remain with long-term efficacy
BRAF Inhibitors (e.g., Dabrafenib)	BRAF mutations in tumor cells	Small molecules that inhibit mutant BRAF signaling pathway in NSCLC tumors	1. Effective in BRAF V600E-mutant NSCLC 2. Combined with MEK inhibitors, shows synergistic effect	1. Limited to BRAF-mutant tumors2. Potential for drug resistance	FDA-approved for BRAF V600E-mutant NSCLC
Targeted HER2 Therapies (e.g., Trastuzumab)	HER2 overexpression in tumor cells	Monoclonal antibodies targeting HER2 receptor to block signaling pathways involved in tumor growth	1. Effective in HER2-overexpressing NSCLC 2. Synergistic effect when combined with chemotherapy	1. Limited to HER2-positive tumors2. Resistance over time	Investigational in HER2-positive NSCLC, ongoing clinical trials
NTRK Inhibitors (e.g., Larotrectinib)	NTRK gene fusions in tumor cells	Small molecules targeting NTRK gene fusions to inhibit downstream oncogenic signaling pathways	1. Effective in tumors with NTRK fusions2. Rapid tumor shrinkage observed in NTRK fusion-positive tumors	1. Limited to NTRK-fusion-positive tumors2. Resistance may occur	FDA-approved for NTRK fusion-positive tumors across various cancers, including NSCLC
MEK Inhibitors (e.g., Trametinib)	MEK pathway in tumor cells	Small molecules that inhibit MEK in the MAPK signaling pathway, disrupting tumor cell proliferation	Effective in combination with BRAF inhibitors2. Synergistic effects in BRAF-mutant NSCLC	1.Side effects like rashes and diarrhea 2. Limited efficacy outside BRAF-mutant tumors	FDA-approved in BRAF-mutant NSCLC as part of combination therapy with BRAF inhibitors

An important aspect of CD44-targeted nanocarriers is their ability to potentially enhance the effectiveness of current targeted treatments via synergistic combinations (17). Merging CD44 targeting with medicines focused on separate molecular markers or pathways, such as EGFR or VEGF, there exists the potential of establishing synergistic effects (130). This strategic merger targets many mechanisms involved in the growth of NSCLC, providing a chance to improve treatment results. CD44-targeted nanocarriers provide a significant benefit by specifically targeting the desired area, therefore reducing the risk of general toxicity compared to treatments that are not targeted (119). This is especially noteworthy when compared to traditional chemotherapy, which is well-known for its adverse effects on healthy tissues. Targeting CD44 with increased specificity minimizes the likelihood of off-target effects, providing a safer and better-tolerated treatment choice (131).

Nevertheless, despite these benefits, the incorporation of CD44-targeted nanocarriers into NSCLC therapy presents several obstacles. The variation in CD44 expression across patients with NSCLC is a challenge that has to be overcome by implementing techniques that may effectively cater to the different patient groups (132). Moreover, the ever-changing and intricate tumor microenvironment presents challenges in the movement of nanocarriers and the administration of drugs, necessitating thoughtful deliberation. Ultimately, the examination of CD44-targeted functionalized nanocarriers in relation to other targeted therapeutics for NSCLC therapy highlights their distinct characteristics, possible collaborations, and obstacles (127). The adaptability of their targeting strategy, combining potential with current treatments, decreased overall toxicity, and precise targeting emphasizes the potential of CD44-targeted nanocarriers in the developing field of tailored therapy for NSCLC.

## 7 Future prospective: potential advancements and innovations in CD44-targeted nanocarriers for NSCLC therapy

The future of NSCLC treatment holds great promise for revolutionary breakthroughs and developments in CD44-targeted nanocarriers (133). Combining CD44 targeting with other treatment approaches, such as immunotherapy or radiation, has the potential to provide synergistic benefits, simultaneously addressing many aspects of NSCLC development (134). One promising aspect in the future of CD44-targeted nanocarriers is the development of delivery systems that can detect and adjust to changing circumstances inside the tumor microenvironment. This novel method has the potential to completely transform the way drugs are delivered by allowing tiny carriers to release therapeutic substances in response to precise signals inside malignant tissue, therefore maximizing the effectiveness of therapy (135). In addition, in the future, there may be a merging of improved imaging techniques with CD44-targeted nanocarriers. This integration enables both real-time monitoring of medication distribution and the acquisition of significant information about the geographical and temporal dynamics of nanocarriers inside the tumor. This contributes to a more thorough knowledge of how these nanocarriers behave. An innovative part of future research is the development of customized therapeutics specifically designed to match the unique characteristics of each patient. By using the molecular and genetic properties, nanocarriers that specifically target CD44 might be engineered to deliver medicines with increased effectiveness and fewer negative side effects (136). This patient-centric approach is in line with the main objective of precision medicine, guaranteeing that treatments are tailored to the unique biology of each patient.

However, there are still ongoing obstacles despite the promising opportunities. The varied expression of CD44 across NSCLC patients is a substantial challenge that necessitates sophisticated approaches for successful targeting across different patient groups (25). Thorough investigations assessing the enduring safety and effectiveness of CD44-targeted nanocarriers are essential for their successful use in clinical settings. The transition from encouraging preclinical findings to regulatory approval requires diligent focus on specifics and comprehension of the many biological intricacies involved. Furthermore, future studies need to give priority to outcomes that are centered on the patient, including factors such as the quality of life, the capacity to tolerate therapy, and the total length of living. Integrating patient-reported outcomes into clinical trials might provide a viewpoint on the influence of CD44-targeted nanocarriers on people receiving NSCLC medication, guaranteeing that progress in treatment leads to significant enhancements in patients’ well-being. Ultimately, the prospects for CD44-targeted nanocarriers in NSCLC treatment are very promising and evolving, offering several possibilities for future improvements and enhancements. The future trajectory of this revolutionary profession will heavily rely on achieving a careful equilibrium between technical innovation, thorough research to tackle current difficulties, and a steadfast dedication to enhancing patient outcomes.

There is a promising opportunity to investigate combination treatments that use nanocarriers targeting CD44. The integration of these nanocarriers with conventional chemotherapeutics, immunotherapies, or targeted medicines has the potential to enhance treatment outcomes by synergistically combining their effects, therefore offering a more holistic approach to managing NSCLC. The long-term consequences of CD44-targeted nanocarriers on survivors of NSCLC have not been extensively investigated. Research should prioritize the investigation of late-onset side effects, the development of resistance mechanisms, and the impacts on overall survival and quality of life. This will ensure a thorough evaluation of the therapy’s consequences.

Interdisciplinary cooperation is crucial for advancing the limits of understanding and promoting advancements in research on CD44-targeted nanocarriers for NSCLC. Partnerships with specialists in biomedical engineering and material science have the potential to accelerate the development of innovative nanocarrier platforms. Nanomaterial advancements, drug delivery system improvements, and surface alterations have the potential to increase the specificity, stability, and therapeutic payload of nanocarriers that target CD44. To comprehend the complex interaction between CD44 and the immune system, it is essential to engage in cooperation with experts in immunology and cancer biology. Investigating the ability of nanocarriers targeted at CD44 to modulate the immune system and examining their influence on immune responses against tumors might lead to the development of more efficient immunotherapeutic approaches.

It is very necessary to have a strong partnership with clinical researchers and oncologists to effectively apply laboratory discoveries to real-world medical treatment. It is essential to conduct carefully planned clinical studies to evaluate the safety and effectiveness of CD44-targeted nanocarriers in various groups of patients. This is necessary to confirm their potential as treatment. Engaging patient advocacy organizations in research endeavors guarantees a patient-centric approach. These partnerships may provide valuable insights into the views, preferences, and goals of patients, which can have a significant impact on the design of clinical trials and enhance the entire patient’s experience with CD44-targeted nanocarrier medicines. Ultimately, the potential of CD44-targeted nanocarriers in treating NSCLC depends on the exploration of new and unexplored areas, as well as the promotion of cooperation across different disciplines. Researchers may advance the development of better, customized, and patient-friendly treatment approaches for NSCLC by filling information gaps, adopting breakthrough technologies, and working together across many disciplines. The collaboration of knowledge and skills from many fields is crucial in unlocking the capabilities of CD44-targeted nanocarriers and revolutionizing the landscape of NSCLC therapy.

## 8 Conclusion

In conclusion, the investigation of CD44-targeted functionalized nanocarriers for NSCLC is a compelling account of advancements and breakthroughs in cancer treatment. The upregulation of CD44 in NSCLC has played a crucial role in developing the development of nanocarriers, offering a focused strategy for drug administration that shows the potential to surpass the constraints of traditional therapies. The examination of CD44 as a biomarker revealed its diverse involvement in NSCLC, including its molecular attributes, its correlation with cancer stem cells, and its prognostic significance. The fundamental knowledge served as the basis for the careful development and production of nanocarriers, using a range of ligands such as hyaluronic acid, aptamers, and antibodies. The evaluation of nanocarrier characteristics, including dimensions, morphology, and electrostatic potential, highlights the complexities associated with optimizing interactions with CD44 receptors to improve the accuracy of drug delivery. This review aims to facilitate ongoing research and cooperation by connecting molecular insights, nanocarrier design, and translational advancements. The goal is to contribute to the development of improved and targeted therapies for patients with NSCLC.
